# Brain States That Encode Perceived Emotion Are Reproducible but Their Classification Accuracy Is Stimulus-Dependent

**DOI:** 10.3389/fnhum.2018.00262

**Published:** 2018-07-02

**Authors:** Keith A. Bush, Jonathan Gardner, Anthony Privratsky, Ming-Hua Chung, G. Andrew James, Clinton D. Kilts

**Affiliations:** ^1^Brain Imaging Research Center, University of Arkansas for Medical Sciences, Little Rock, AR, United States; ^2^College of Medicine, University of Arkansas for Medical Sciences, Little Rock, AR, United States

**Keywords:** brain state, affect, classification, inter-study reproducibility, IAPS, MVPA

## Abstract

The brain state hypothesis of image-induced affect processing, which posits that a one-to-one mapping exists between each image stimulus and its induced functional magnetic resonance imaging (fMRI)-derived neural activation pattern (i.e., brain state), has recently received support from several multivariate pattern analysis (MVPA) studies. Critically, however, classification accuracy differences across these studies, which largely share experimental designs and analyses, suggest that there exist one or more unaccounted sources of variance within MVPA studies of affect processing. To explore this possibility, we directly demonstrated strong inter-study correlations between image-induced affective brain states acquired 4 years apart on the same MRI scanner using near-identical methodology with studies differing only by the specific image stimuli and subjects. We subsequently developed a plausible explanation for inter-study differences in affective valence and arousal classification accuracies based on the spatial distribution of the perceived affective properties of the stimuli. Controlling for this distribution improved valence classification accuracy from 56% to 85% and arousal classification accuracy from 61% to 78%, which mirrored the full range of classification accuracy across studies within the existing literature. Finally, we validated the predictive fidelity of our image-related brain states according to an independent measurement, autonomic arousal, captured via skin conductance response (SCR). Brain states significantly but weakly (*r* = 0.08) predicted the SCRs that accompanied individual image stimulations. More importantly, the effect size of brain state predictions of SCR increased more than threefold (*r* = 0.25) when the stimulus set was restricted to those images having group-level significantly classifiable arousal properties.

## Introduction

Core affect is a central construct in our understanding of emotion (Russell and Barrett, 1999), and its roles in situationally-appropriate behavior (Gross, 2015) and self-preservation (Plutchik, 2001). Moreover, functional magnetic resonance imaging (fMRI) blood oxygen-level dependent (BOLD) signal has consistently identified brain nodes and neurocircuits that are activated in response to affective and emotional stimulation (Bush et al., 2000; Killgore and Yurgelun-Todd, 2007; Gerber et al., 2008; Wager et al., 2008; Hagan et al., 2009; Posner et al., 2009; Colibazzi et al., 2010; Lindquist et al., 2012, 2016). More recently, multivariate pattern analysis (MVPA) of BOLD responses (Haxby et al., 2001), such as machine learning-based neural activation pattern classification of affective stimuli, has been deployed to overcome statistical limitations of canonical univariate analysis (Habeck and Stern, 2010), common to early fMRI analyses of emotion processing (Hamann, 2012), and has resulted in improved classifier performance (Haynes and Rees, 2006; Norman et al., 2006; O’Toole et al., 2007). Indeed, multivariate analysis has significantly advanced our understanding of the neurobiological bases of affect and emotion processing (Pessoa and Padmala, 2007; Ethofer et al., 2009; Peelen et al., 2010; Said et al., 2010; Sitaram et al., 2011; Kassam et al., 2013).

MVPA of fMRI response is based on the brain state hypothesis of cognitive processing: that there exists a one-to-one mapping between a brain state (i.e., a temporally succinct pattern of distributed neural activations) and the cognitive process that this state encodes. This hypothesis is particularly relevant to past MPVA-based attempts to classify brain states induced by visual stimuli according to the normed affective content of the stimuli across both discrete emotions (Saarimäki et al., 2016) and the independent valence and arousal properties of dimensional emotion (Baucom et al., 2012; Bush et al., 2017). The brain states induced within these studies exhibited patterns of neural activation that were distributed widely throughout the cortex and subcortex (Chang et al., 2015; Saarimäki et al., 2016; Bush et al., 2017) and challenged earlier hypotheses that assigned specific neuroanatomical loci to each discrete emotion (Ekman, 1999; Izard, 2011).

Though multivariate analyses of affective brain states have rapidly emerged, it remains important to inform and interpret classification outcomes based on how these states are induced, how these states generalize across approaches and studies, the fidelity by which these states capture affect processing both within and across subjects, and how these units of neural information processing explain independent measurements and properties of affect. Critically, classification accuracy varies widely between fMRI-based affective perception studies, despite their use of comparable methodology; this variance weakens the brain state hypothesis and suggests the existence of one or more unaccounted sources of variation in MVPA of affect processing. Drawing upon learned lessons from the literature of univariate analysis of affect processing (Vytal and Hamann, 2010; Lindquist et al., 2012), it is incumbent on the field of affective neuroscience to identify, understand and control the sources of inter-study differences.

The goal of this fMRI study was to explore the brain state hypothesis of affect processing by assessing the inter-study encoding reliability for similar but independent affective perception experiments, the impact of affect stimulus selection on the accuracy of brain state classification across affective properties of valence and arousal, and to assess independent psychophysiological support for the existence of affect processing brain states. Here we attempted to validate brain state predictions for a measure of emotional arousal of the autonomic nervous system (ANS) related to the skin conductance response (SCR) (Bradley et al., 2001).

**Figure 1:**
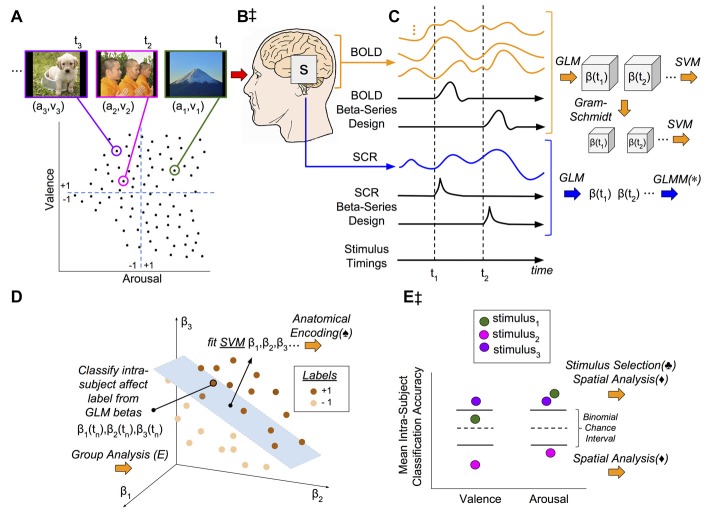
Methodological and conceptual overview. **(A)** Experiment design: 90 image stimuli were selected from the International Affective Picture System (IAPS) such that the image subset represented the full range of the continuously-valued component properties, valence (v) and arousal (a). Closed circles represent affective coordinates of image stimuli. Dashed lines represent the Likert scores representing the theoretical dividing line between positive (+1) and negative (−1) affect classes in the arousal-valence plane. The images were presented for 2 s interleaved with random inter-trial intervals (ITIs) (2–6) s. **(B,‡)** Signal acquisition: image presentations were concurrent with functional magnetic resonance imaging (fMRI) to measure blood oxygen level dependent (BOLD) response as well as skin conductance response (SCR). **(C)** Brain and psychophysiological state estimation: (1) fMRI signals were preprocessed to remove noise and motion artifacts and segmented to remove all voxels except gray matter (GM); (2) SCR signals were preprocessed to remove noise and tonic signal components; (3) for each stimulus, neural activation patterns were extracted via the beta-series method (Rissman et al., 2004); and (4) neural activation patterns (originally on the order of 40,000–50,000 dimensions) were subsequently reduced to 90-dimensions according to the method of Gram-Schmidt (GS) orthonormalization (GS). **(D)** Classification of affective signals: The individual patterns of image stimulus-related neural activation, each matched to the labels of the stimulus from which they were derived (see panel **A**), were used to conduct intra-subject leave-one-out-cross-validated (LOOCV) linear support vector machine (SVM) classification. In the example shown, the hyperplane is used to classify the affective class (i.e., +1 or −1) of a novel response point (the neural activations induced by the *n*th stimulus). **(E,‡)** Conceptual model: We hypothesis that a brain state, s (see panel **B**), simultaneously encodes both the dimensional affective properties of each individual image stimulus as well as its psychophysiological response. Thus, brain states (see panel **C**) should accurately classify affective labels (see panel **E**, stimulus_3_) and predict SCRs (lower half of panel **C**). Group-level classification error for each stimulus for each affective property can be attributed to one of two sources: (1) the stimulus induces brain states that inconsistently encode the conveyed affect (either through weak effect-size or wide variance; see panel **E** classification of valence, stimulus_1_); or, (2) the stimulus consistently induces brain states that are incongruent with the normative affective rating of the stimulus (see panel **E**, stimulus_2_). (*) General linear mixed-effects models quantify the SVM prediction of SCR vs. the observed SCR. (♠) The individual SVM models are transformed (Haufe et al., 2014) into encoding representations of affect state and anatomically analyzed group-wise (not pictured). (♣) Visual stimuli are selected for evaluation of factors confounding intra-subject classification performance if they exceed (correct) and subceed (incorrect) chance-levels of accuracy. (⧫) Performance-selected stimuli are analyzed for spatial patterns within the affective coordinate space (lower half of panel **A**). (Note) The specific points, time-series, and classification models presented in this figure are for illustrative purposes only; they are intended to approximate data properties within the experiment, but they do not represent real or observed data.

In pursuit of these study goals, we conducted an analysis of data for an image-based affect perception experiment related to concurrent fMRI and SCR measurements based on the methodological design and conceptual framework depicted in Figure 1. Our study found that this methodological approach to deriving affective brain states from fMRI yielded brain states that are highly consistent between two unique fMRI studies, acquired on the same scanner (more than 4 years apart) but involving unique subjects and image stimuli. Moreover, we identified a significant influence of affect stimulus selection in determining the accuracy of classification of perceived affect state from fMRI-derived brain states. Indeed, the relationship between classifier performance and stimulus was conditional based on the self-reported affective ratings of the stimuli. Individual stimuli were found to both significantly improve or reduce classification performance; moreover, the individual predictability of stimuli translated into SCR prediction performance, suggesting a direct mechanistic connection between brain state and autonomic arousal.

## Materials and Methods

### Study Overview

We conducted analyses of data acquired from the Intrinsic Neuromodulation of Core Affect (INCA) study, a functional neuroimaging exploration of emotion perception, unguided emotion regulation, and real-time fMRI guided emotion regulation. All study procedures were conducted in the Brain Imaging Research Center (BIRC) at the University of Arkansas for Medical Sciences (UAMS). This study was carried out in accordance with the recommendations of the human research policy of the UAMS Institutional Review Board with written informed consent from all subjects. All subjects gave written informed consent in accordance with the Declaration of Helsinki. The protocol was approved by the UAMS Institutional Review Board.

Study participation was conducted in two sessions on separate days. Session 1 included obtaining written informed consent, determining if subjects met clinical exclusionary criteria via structured clinical interview (SCID-I/NP), and administering behavioral surveys and questionnaires. Session 2 included the neuroimaging session, lasting approximately 1 h and comprised of three sequentially administered tasks: System Identification, Resting State and Intrinsic Neuromodulation. This analysis only includes data captured during the System Identification Task.

### Subjects

Twenty subjects completed the System Identification task of the INCA study. After study closure, a retrospective self-audit revealed that one subject met exclusionary criteria (DSM-IV threshold for PTSD and GAD) leading to that subject’s removal from analysis. The participant sample (*n* = 19) used for this analysis had the following demographic characteristics: age (mean (SD)): 28.2 (9.2), range 20–56; sex: 10 (52.6%) female; race/ethnicity: 16 (84.2%) self-reporting as White or Caucasian, 2 (10.5%) as Black or African-American, 1 (5.3%) as Hispanic or Latino; education (mean (SD)): 17.1 (2.1) years, range 14–21; IQ (mean (SD)): 107.4 (15.0), range 81–137. All subjects were right-handed, native-born United States citizens (a control for the applicability of imageset normative scores), medically healthy, with no current psychopathology, no current usage of psychotropic medication, and produced a negative urine screen for drugs of abuse immediately prior to the MRI scan. Additionally, all subjects’ vision was corrected to 20/20 during the MRI scan and color-blindness was exclusionary.

### System Identification Task

Image stimuli drawn from the International Affective Picture System (IAPS) (Lang et al., 2008), a widely-cited normed imageset that has been used in two prior MVPA-based studies of the classification of perceived affect (Baucom et al., 2012; Bush et al., 2017), were presented using two randomly interleaved formats, extrinsic (imageset A) and intrinsic (imageset B). These formats are distinguished by the instructions to either passively view (extrinsic) or actively experience (intrinsic) the affective content of the IAPS stimulus. The extrinsic format presented an image for 2 s (stimulation) succeeded by a fixation cross for a random inter-trial interval (ITI) sampled uniformly from the range of 2–6 s. The intrinsic format is multi-part: (1) it presented an image for 2 s; (2) a visual cue (the word “FEEL”) is superimposed over the image for 2 s to instruct the participant to anticipate the attempt to volitionally re-experience the affect state portrayed by the image; (3) the image disappeared leaving the visual cue for 10 s during which the participant actively attempted to volitionally re-experience the image’s affective content; and (4) a fixation cross appeared for an ITI sampled uniformly from the range of 2–6 s (*μ*_ITI_ = 4.16 s, σ_ITI_ = 1.13 s).

IAPS image presentations were balanced across two 9.25 min scans according to the images’ normative valence and arousal scores. Within each scan, extrinsic and intrinsic formats were temporally arranged such that: (2) no more than three consecutive intrinsic formats appear during a scan; (1) each scan must begin with an extrinsic format; (3) all scans begin and end with positive valence images; and (4) all pairwise GLM regressors constructed from the stimulus timings via the canonical hemodynamic response (HRF) function were correlated less than 0.25. Note, these discrete categories of regressors (positive valence, negative valence, high arousal, and low arousal) were derived from each image’s normative Likert score relative to the middle Likert score (5). Experimental designs (image order and ITIs) were sampled uniformly randomly for each scan until a simulated design simultaneously fulfilled all four criteria. The design was then fixed for all subjects. The analysis presented here includes data captured during extrinsic format presentation only.

### Image Stimuli Selection

Stimulus imageset A consisted of 90 color IAPS images depicting a broad range of emotional content (e.g., aggression, accidents, injury, social scenes, inanimate objects) drawn from the IAPS imageset. The IAPS reports associated normative scores (mean and standard deviations based upon measurements from a 9-point Likert scale) of image valence (v) and arousal (a). Images were computationally selected from the full IAPS imageset according to a maximum separation heuristic (see Figure 1A). Each newly selected image’s normative valence and arousal scores exhibited the maximum summed Euclidean distance (measured in the arousal-valence plane) relative to the scores of all images currently in the selected imageset. This computational sampling approach ensured that the sampled imageset exhibited the full dynamic range of stimulus intensities for each property, irrespective of stimulus type, available within the full IAPS imageset. An additional 30 unique images (imageset B), selected similarly, were subsequently drawn from the IAPS imageset (not shown in Figure 1A). Note, using the default algorithm, two female (and zero male) erotica images were consistently sampled from the IAPS imageset due to the distribution of these image types within the arousal-valence plane. During debriefings of a pilot phase of this experiment, participants indicated that this discrepancy was distracting. In response, two male erotica images were randomly selected from the IAPS imageset to ensure the presence of an equal number of male and female erotica images in the full image subset; heuristic selection commenced subsequent to this image seeding to construct the imageset used in this study.

### MR Image Acquisition

We acquired imaging data using a Philips 3T Achieva X-series MRI scanner (Philips Healthcare, Eindhoven, Netherlands). Anatomic images were acquired with a MPRAGE sequence (matrix = 256 × 256, 220 sagittal slices, TR/TE/FA = shortest/shortest/8°, final resolution = 0.94 × 0.94 × 1 mm^3^. Functional images were acquired using a 32-channel head coil with the following EPI sequence parameters: TR/TE/FA = 2000 ms/30 ms/90°, FOV = 240 × 240 mm, matrix = 80 × 80, 37 oblique slices, ascending sequential slice acquisition, slice thickness = 2.5 mm with 0.5 mm gap, final resolution 3.0 × 3.0 × 3.0 mm^3^. Parameters for the 32-channel coil were selected to reduce orbitofrontal cortex signal loss due to sinus artifact.

### MR Image Preprocessing

We conducted all MRI data preprocessing via AFNI (Version AFNI_16.3.20; Cox, 1996) unless otherwise noted. Anatomic data underwent skull stripping, spatial normalization to the icbm452 brain atlas, and segmentation into white matter (WM), gray matter (GM), and cerebrospinal fluid (CSF) with FSL (Jenkinson et al., 2012). Functional data underwent despiking; slice correction; deobliquing (to 3 × 3 × 3 mm^3^ voxels); motion correction (using the 10th volume); transformation to the spatially normalized anatomic image; regression of mean time course of WM mask (two voxel eroded), mean time course of CSF mask (one voxel eroded) and 24 motion parameters (Power et al., 2014, 2015); spatial smoothing with a 6-mm FWHM Gaussian kernel; and, scaling to percent signal change. BOLD volumes exhibiting framewise displacement (FD) exceeding 0.5 (Power et al., 2012) were excluded from all subsequent analyses.

### Gray Matter (GM) Masking

We created individual GM masks directly via anatomical segmentation (see “MR Image Preprocessing” section). To conduct group-level analysis of predictive neuroanatomical activations, we constructed a single group-level GM mask by voxel-wise thresholding over all individual GM segmentation masks using an inclusion threshold of 50%, i.e., only voxels identified as GM for at least half of subjects were identified as group-level GM and included in the mask.

### BOLD Beta-Series Construction

We exploited beta-series neural activation patterns to conduct MVPA, similar to prior MVPA applications in affective response modeling (Bush et al., 2017). Beta-series (Rissman et al., 2004) were extracted from fMRI BOLD signal as follows. We concatenated the two runs of the System Identification task into a single fMRI signal. We then constructed the general linear model (GLM) using 3dDeconvolve modified by the -stim_times_IM flag in combination with stimulation times of both image presentation formats (both extrinsic and intrinsic) and the BLOCK4(2,1) model of the hemodynamic response function. Drift artifact models were introduced into the GLM via the -polort A and -concat flags. The 24-dimensional motion model (Power et al., 2014, 2015) was also re-included in the GLM (initially regressed out during preprocessing). Frame-wise displacement censoring was also included using the -censor flag. We subsequently solved the resultant GLM via 3dlss. We then sub-selected only the beta-series corresponding to the extrinsic image presentation format.

### BOLD Beta-Series Dimensionality Reduction

Dimensionality reduction is often an important component of effective MVPA. Past BOLD-based MVPA has focused on voxel-wise dimensionality reduction using a technique that exploits voxel stability across repeated trials of the same class (Mitchell et al., 2008; Shinkareva et al., 2008). However, the stimuli selected for this experiment continuously vary over the label space (i.e., dimensions of affect), which precludes application of the voxel stability approach. Instead, we employed Gram-Schmidt (GS) orthogonalization (Kirby, 2001) to first construct a minimum dimensional orthonormal basis in the subspace spanned by our neural activation space followed by projection of our beta-series into the resultant coordinate system. This lossless compression technique reduced the dimensionality of the beta-series from that of the subjects’ GM masks (typically on the range of 40,000–50,000 voxels) to exactly 90-dimensions, one for each extrinsic format image presentation trial.

### Psychophysiology Data Acquisition

We recorded psychophysiological response measures using a BIOPAC MP150 Data Acquisition System (BIOPAC Systems Inc., Goleta, CA, USA) using the AcqKnowledge software platform for simultaneous recording of skin conductance (via the EDA100C-MRI module), heart rate (via the TSD200-MRI pulse plethysmogram), and respiration rate (via the TSD221-MRI respiration belt) within the MRI environment. SCR recording electrodes were placed on the medial portions of the thenar and hypothenar eminences of the left hand; a ground electrode was placed on the ventral surface of the left wrist. The pulse plethysmogram was placed on the left index finger. The respiration belt was fit over the xiphoid process. All physiological signals were sampled at 2000 Hz.

### Skin Conductance Response (SCR) Preprocessing

Nonlinear signal drift and phasic nuisance artifacts are common in SCR data due to subject motion, individual variability in physiological properties (Lykken and Venables, 1971), and background electromagnetic field instability (Lagopoulos et al., 2005). To minimize the effect of these artifacts on stimulus-evoked SCR measurements, we filtered SCR data according to validated methods (Bach et al., 2009, 2013; Staib et al., 2015): (1) a 10 ms median filter smoothed the data by setting individual SCR samples to the median of data in the preceding and following 10 ms; (2) initial SCR signals were centered at zero by subtracting the mean of the first 10 ms of data from the SCR dataset. This zeroing of initial data prevents signal distortion by the third filtering stage; (3) the data were bandpass filtered using a first-order bi-directional Butterworth filter in the frequency between 0.033 Hz and 5 Hz, passing waveforms between 0.2 s and 30.3 s and removing slow trends in data drift to optimize the passage of stimulus-evoked SCRs that universally follow a waveform of approximately 30 s; (4) data were downsampled to 10 Hz; and (5) *z*-scored within runs to remove inter-subject variance in SCR amplitudes due to peripheral factors. Half of the SCR data (i.e., one of two runs each) from three subjects was excluded from analyses as these subjects did not display a measurable SCR or data acquisition was corrupted. These exclusions (7.9% of all data) were well below standard SCR exclusion rates (Kredlow et al., 2017).

### Construction of SCR Beta-Series

Analogous to the feature space construction from BOLD signal, beta-series (Rissman et al., 2004) were also extracted from SCR signal. Due to subtle differences in the nature of the signal and its preprocessing steps, we employed an alternate processing pipeline custom-implemented in Matlab (The Mathworks Inc., 2017) and comprised of the following steps: (1) based on the stimulation times of all image presentation formats (both extrinsic and intrinsic) and the SCRalyze library’s canonical SCR function (Bach et al., 2010), we constructed a beta-series design function; (2) we then filtered this design function identically to the SCR data (above) to account for peak-shifting; (3) we z-scored the resulting individual design vector; and (4) we solved for the beta-series via the regstats function. We then sub-selected only the beta-series corresponding to the extrinsic image presentation format.

### Multivariate (i.e., Multivoxel) Pattern Analysis (MVPA)

MVPA was conducted via linear support vector machine (SVM) for both classification (Boser et al., 1992) and regression (Vapnik, 1995) using the implementation found within the Matlab Statistics Toolbox (regression used fitrsvm and classification used fitcsvm) and default parameters (available on-line). MVPA predictions based on beta-series were conducted subject-wise according to the convention, ${\text{y}}_{\text{i,j}}=\text{f(}\beta_{\text{i,j}}^{\text{P}}\text{)}$, where f denotes the trained SVM classification or regression model (see “Affect Classification Intra-subject Training and Cross-Validation” and “SCR Regression Intra-subject Training and Cross-Validation” sections), y_i,j_ is the predicted outcome for subject, i, and stimulus, j; $\beta_{\text{i,j}}^{\text{P}}$ is the P-set of betas, P Є {GM, GS, SCR}, where GM denotes BOLD signal betas for all GM voxels, GS denotes BOLD signal betas for all GS dimensions, and SCR denotes SCR signal betas. We conducted the following predictions:

(1) $${\text{\textasciitilde{}v}}_{\text{i},\text{j}}\left(\text{GS}\right)=\text{f}\left(\beta_{\text{i},\text{j}}{}^{\text{GS}}\right),$$

(2) $${\text{\textasciitilde{}a}}_{\text{i},\text{j}}\left(\text{GS}\right)=\text{f}\left(\beta_{\text{i},\text{j}}{}^{\text{GS}}\right), and$$

(3) $$\sim \beta_{\text{i},\text{j}}{}^{\text{SCR}}\left(\text{GS}\right)=\text{f}\left(\beta_{\text{i},\text{j}}{}^{\text{GS}}\right),$$

where ~y_i,j_(·) is the prediction for subject, i, and stimulus, j, based upon beta-series (·); *v* denotes the binary valence label +1, −1; and, *a* denotes the binary arousal label +1, −1. We also conducted the following validation predictions to neuroanatomically assess the learned hyperplanes:

(4) $${\text{\textasciitilde{}v}}_{\text{i},\text{j}}\left(\text{GM}\right)=\text{f}(\beta_{\text{i},\text{j}}{}^{\text{GM}}) and$$

(5) $$\sim {\text{a}}_{\text{i},\text{j}}\left(\text{GM}\right)=\text{f}\left(\beta_{\text{i},\text{j}}{}^{\text{GM}}\right).$$

### Affect Classification Intra-subject Training and Cross-Validation

MVPA classification accuracy of affective property labels from fMRI beta-series (Equations 1 and 2) was cross-validated stimulus-wise within each subject, i.e., intra-subject leave-one-out cross-validation (LOOCV). Therefore, within each subject, i, for each image stimulus, j (i.e., the beta activation and label forming the test set, S_tst_), the disjoint set of (*n* = 89) stimuli form the beta activations and labels of the initial training set. The initial training set beta activations were subsequently divided into two subsets: beta activations associated with positive class labels, denoted subset L+, and beta activations associated with negative class labels, denoted subset L−. Note that labels depend on the affective property being classified (either valence or arousal). Due to the arrangement of normative scores of the stimulus set (see Figure 1A), the sizes of these subsets may be imbalanced. Therefore, the smaller of these two subsets, S_min_, was identified (|S_min_| = N_min_) and, subsequently, N_min_ elements of the larger subset, S_max_, were uniformly randomly sampled, forming a third subset, S_rdx_ (|S_rdx_| = N_min_). Subsets S_min_ and S_rdx_ were then combined to form a final training dataset, S_trn_ (|S_trn_| = 2 · N_min_), having equal numbers of positive and negative class labels (ensuring that the null hypothesis is truly 0.5 probability of assigning the positive label, +1). The SVM was then fit to S_trn_ and applied to predict the label of S_tst_. Each prediction was individually stored for subsequent analysis. Because the training subset incorporates random sampling from subset S_max_, we control for sampling effects by repeating the entire cross-validation process 30 times for each subject, and report for each subject the mean LOOCV classification accuracy over these repetitions.

### Anatomical Representation of Affective Encoding

We projected each intra-subject SVM hyperplane to its encoding representation via the Haufe-transform (Haufe et al., 2014; Hebart et al., 2015). Then for each voxel in the group-level GM mask, we calculated the group-level mean and group-level distribution of encodings (one-sample *t*-scores), respectively for valence and arousal.

### Encoding of Affective Pictures (EAP) Mean Hyperplane Construction

The mean hyperplanes encoding valence and arousal reported in Bush et al. (2017) were Haufe-transformations of the mean inter-subject decoding hyperplanes. This was deemed inappropriate for direct comparison to the mean hyperplanes in this work because the impacts of the order of averaging and Haufe-transformation were difficult to estimate. Rather, we applied the encoding estimation methodology presented in this work to the hyperplanes fit to the EAP study’s data; specifically, we computed the Haufe-transform for each inter-subject cross-validated hyperplane, and then averaged the encoding hyperplanes to form the mean (i.e., the mean of the encodings rather than the encoding of the mean).

### SCR Regression Intra-Subject Training and Cross-Validation

MVPA regression of SCR beta-series from fMRI GS beta-series (Equation 3) was cross-validated stimulus-wise within each subject, i.e., intra-subject LOOCV. Therefore, within each subject, i, for each image stimulus, j (i.e., the fMRI GS beta-series neural activation and SCR beta-series label forming the test set, S_tst_), the disjoint set of fMRI GS beta-series neural activations and SCR beta-series labels form the training set, S_trn_. The SVM regression model is trained on S_trn_ and applied to predict the label of S_tst_. Each of these predictions was individually stored for subsequent analysis.

### Intra-Subject Stimulus Subset Selection

Arguably, the largest source of inter-study variation in MVPA-based classification of affect perception is the method of stimulus-driven implicit affect processing (i.e., stimulation). Stimulation task modalities span affective words and video clips (Saarimäki et al., 2016), voice-derived audio (Ethofer et al., 2009), facial expressions (Pessoa and Padmala, 2007), as well as complex imagery (Baucom et al., 2012; Bush et al., 2017). Beyond stimulus modality itself, however, the distribution of affective properties within the modality (or even within a specific dataset of a specific modality) may critically impact stimulus-driven responses and, consequently, the inferences drawn from the induced brain states. Baucom et al. (2012) selected IAPS image stimuli from four highly focused clusters within the arousal-valence plane based on their maximal separation. In contrast, our image stimuli are algorithmically selected to span the entire IAPS arousal-valence plane (see Figure 1A) by maximizing the separation between individual images. This difference suggests an important methodological consideration in stimulus-induced affect processing, which we explored.

To evaluate the reliability of image stimulus-induced affect processing, we conducted subject-specific stimulus selection based on group-level SVM classification consistency as follows. For each subject, i, for each stimulus, j, we evaluated the reliability of stimulus j based on the distribution of predictions made for this stimulus by the disjoint set of study subjects (*n* = 18). Each stimulus that exhibited prediction accuracy greater than chance (binomial distribution, *n* = 18, *p* = 0.5, *α* = 0.05) was identified as “reliable” and added to the subject’s reliable stimulus set, RSS_i_. Each stimulus that exhibited prediction accuracy worse than chance (binomial distribution, *n* = 18, *p* = 0.5, *α* = 0.05) was identified as “unreliable” and added to the subject’s unreliable stimulus set, USS_i_. Each subject’s RSS and USS accuracies were calculated from the class label predictions made by the subject’s hyperplanes fit to the FSS but restricted only to the predictions for the stimuli within these subsets. Note, a group-level RSS, and a group-level USS, were also formed from the stimuli jointly represented in all subject sets. These group-level sets were used to conduct subsequent analysis of stimulus dependent factors impacting classification performance.

### Simulation of Chance Subset Proportion

We assume that each image stimulus will convey affective information according to a biased coin well-represented by the mean accuracy of our intra-subject classifiers (p(Head) = accuracy), respectively for each affective dimension. Each of the 90 images is assumed independent in this quality as are the 19 subjects. Given this, we can simulate the proportion of stimuli in a set of 90 stimuli that would, by chance, appear to be a reliable stimulus set according to random sampling of this biased coin. We did this by randomly sampling from our coin 19 times for each of the 90 simulated stimuli. We then computed the fraction of the 90 stimuli for which the number of heads exceeds (or subceeds) chance (binomial distribution, *n* = 19, *p* = 0.5, *α* = 0.05). We repeated this simulation 1000 times and computed the probability that random sampling would produce an RSS (or USS) as large or larger than the observed RSS (or USS) set-sizes (bootstrap method test), respectively, for each affective dimension.

### Test of Spatial Multimodality

We hypothesized that the normed affective content of a stimulus should influence its ability to be successfully discriminated by its induced brain state at a group level. However, we also posited that there may be interactions in the arousal-valence plane that cause a single-dimensional analysis to be unreliable. Therefore, as a surrogate to our hypothesis, we assume that reliably discriminated stimuli should cluster within the arousal-valence plane. To test this hypothesis, we measured the multimodality of the RSS according to the Hartigan dip statistic (Hartigan and Hartigan, 1985). For valence- and arousal-derived RSSs, respectively, we computed the dip statistic for the valence and arousal axes. To account for possible arousal-valence interactions, we computed the dip statistic for all rotations of the arousal-valence plane on the range of −π/4 to π/4 radians sampled at increments of 0.01 radians and identified the angle that maximized the dip test. We then performed 1000 bootstraps in which we sampled from the FSS a subset of the points (also rotated according to the maximizing angle) of the same size as the RSS and computed its dip test. We report the *p*-value as the probability of these bootstrap tests having a dip statistic greater than observed dip statistic.

### Mixed Effects Modeling

To measure the predictive effect size of the SVM regression framework, we modeled experimental measurements as functions of predicted measurements via general linear mixed effects model (GLMM). In all experiments, the measure of interest was the experimental measure. The fixed-effect was the predicted measure. Random slope and intercept effects were modeled subject-wise. GLMMs were solved using Matlab’s fitlme function. Effect-size (Pearson’s *r*) was calculated based on the resultant fixed-effects.

### Validation of Dimensionally Reduced Activations

We validated the relationship between multivariate classifications conducted using GS reduced dimensionality beta-series against whole-brain GM voxel-based betas-series via GLMM. The measures of interest were the GM voxel derived SVM hyperplane distances predicted for each activation of the beta-series. The fixed effects were GS derived SVM hyperplane distances predicted for each activation of the beta-series. Random effects were modeled subject-wise. Validations were conducted separately for valence and arousal. Predicted hyperplane distances were shown to be significantly related (see Supplementary Figure S1). Prediction effect sizes were found to be very large for both valence (*r* = 0.86) and arousal (*r* = 0.84).

## Results

### Brain States Exhibit Canonical Functional Neuroanatomical Correlates of Affect Perception

**Figure 2:**
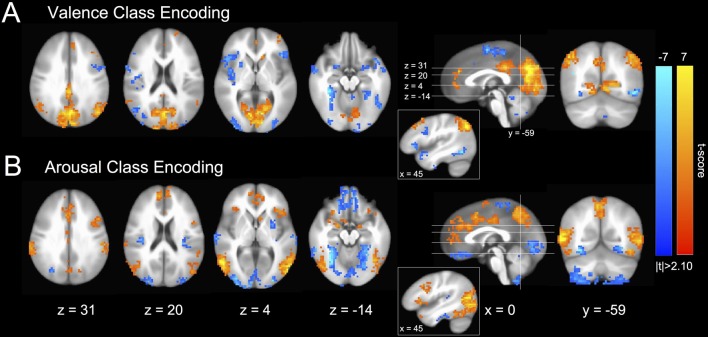
Group-level distributions of GM SVM intra-subject emotion perception encoding parameters (Haufe et al., 2014). **(A)** Valence classification encoding. **(B)** Arousal classification encoding. Colors indicate both the group-level strength of activations (voxel-wise; 2-sided 1-sample *t*-test; null: *μ* = 0) as well as the stimulus class under which voxel activation would be positively increased (warm colors indicate positive valence or high arousal and cool colors indicate negative valence or low arousal). Slices are depicted in Talairach coordinate space and neurological convention (image left equals participant left). Voxel intensities are thresholded at |*t*| > 2.10 with maximum voxel intensity set to |*t*| = 7.0. Only clusters of 20 or greater contiguous voxels (NN = 1) are depicted.

Encoding models of perceived valence and arousal (see Figure 2), transformed from MVPA-based decoding models of fMRI-derived brain states (Haufe et al., 2014), are highly consistent with functional neuroanatomical regions of affect processing within the univariate analysis literature. We observed group activations in the canonical core affect processing regions (bilateral amygdala (amyg); anterior insula (aIns), occipital frontal cortex (OFC), striatum, as well as rostral, dorsal, and middle cingulate cortices (rCC, dCC, mCC); Barrett et al., 2007; Kober et al., 2008). Further, as would be anticipated by our paradigm’s reliance on image-driven neural responses, we observed activation in canonical conceptualization regions (ventral medial prefrontal cortex (vmPFC), dorsal medial PFC (dmPFC), medial temporal lobe (mTL), hippocampus (hipp), and posterior cingulate cortex (pCC); Lindquist et al., 2012). These findings also, in large part, agree with the functional neuroanatomy of affect processing identified in prior MVPA-based emotion classification studies for both the discrete (Saarimäki et al., 2016) and dimensional (Bush et al., 2017) models of emotion, which found that affective perception at the neural processing network level is dominated by bilateral amyg, aIns, vmPFC, dCC, pCC, precuneus, and medial occipital cortex (mOC) activations.

### Brain States Are Reproducible Across Two Independent Studies of Affect Perception

**Figure 3:**
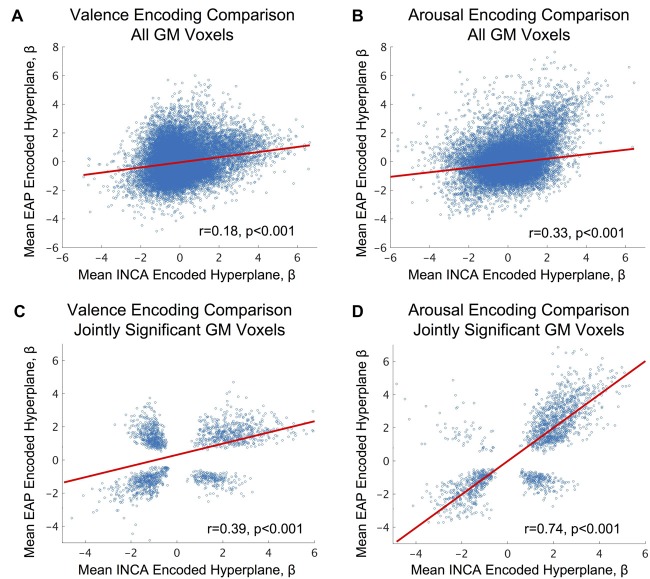
Analysis of fMRI-derived brain states’ inter-study consistency between the current study (Intrinsic Neuromodulation of Core Affect, INCA) and a previous image-based affect perception study (EAP), see Bush et al. (2017) for study details. **(A,B)** fMRI-derived affective encodings (Haufe et al., 2014) identified from the INCA study predicted the affective encodings identified by the EAP study over all jointly identified gray-matter (group-level) voxels across both studies for valence and arousal, respectively. **(C,D)** INCA affective encodings predict affective encodings identified by the EAP study over all jointly identified gray-matter voxels (group-level) that are also jointly significant (2-sided 1-sample *t*-test, *α* = 0.05; null: μ = 0; *N*_EAP_ = 32, *N*_INCA_ = 19) across both studies for valence and arousal, respectively. Blue circles depict the scatter plot of individual voxel comparisons. Red lines depict the robust regression fit to the individual voxels. Effect sizes are reported as Pearson’s *r*. *P*-values refer to the significance of the robust regression linear coefficient (*F*-test, *α* = 0.05).

We conducted a direct comparison of the neural response patterns identified in this study with a similarly designed, but independent, study previously conducted by our lab, entitled EAP. See Bush et al. (2017) for participant, stimuli, and task details of the EAP study, and see “Materials and Methods: EAP Mean Hyperplane Construction” section for details on how the mean encoding parameters were formed from parameters computed for the EAP study. Direct cross-study comparison of the encoding parameters calculated for whole-brain GM voxels (see Figures 3A,B) found significant correlation between the studies with moderate effect sizes. However, we also directly compared encoding parameters between the studies for only those voxels that were conjointly (for both studies simultaneously) and group-wise significantly activated (2-sided 1-sample *t*-test, *α* = 0.05; null: *μ* = 0). Cross-study comparison within this voxel subset, exhibiting both responsiveness to affective content and robustness across subjects, found significant correlations (see Figures 3C,D) of moderately large to very large effect sizes according to standard taxonomy (Cohen, 1992).

### Affect Perception Classification Performance Via fMRI-Derived Brain States Is Affect Stimulus Set-Dependent

Following the methodology outlined in Figures 1A–D (see “Materials and Methods” section for details), we computed stimulus-wise LOOCV classification accuracies over a dataset of 90 IAPS images. Quantified in Table 1, group-level analysis of intra-subject classification accuracy over all 90 IAPS image stimuli (denoted the Full Stimulus Set, FSS) was significantly greater than chance for both valence and arousal, separately, as well as for the classification of overall Affective State (AS) (combined valence and arousal). Quantified subject-wise, we found that 4 of 19 subjects (21%) exhibited significant classification accuracy of the valence property of dimensional affect (null hypothesis is the binomial distribution, *n* = 90 (images), *p* = 0.5, *α* = 0.05) and 11 of 19 subjects (58%) exhibited significant classification accuracy of the arousal property of dimensional affect (null hypothesis is the binomial distribution, *n* = 90 (images), *p* = 0.5, *α* = 0.05).

**Table 1:** Intra-subject classification performance of the reliable stimulus set (RSS), analyzed for group-level significance.

	Valence (V)	Arousal (A)	Affective state (AS)
	Grp. Avg. Acc. (95% CI)	Grp. Avg. Acc. (95% CI)	Grp. Avg. Acc. (95% CI)
Full stimulus set (FSS)	0.56^†^ (0.53, 0.59)	0.61^†^ (0.59, 0.63)	0.34^†^ (0.32, 0.36)
Reliable stimulus set (RSS)	0.85^†‡^ (0.82, 0.88)	0.78^†‡^ (0.74, 0.82)	0.44^†‡^ (0.42, 0.45)

*^†^Finding is significantly greater than chance accuracy (1-sample t-test, *α* = 0.05; null_V,A_: μ = 0.5; null_AS_: μ = 0.25). ^‡^Accuracy is significantly greater than full stimulus set accuracy (2-sided 2-sample t-test, *α* = 0.05; null: *μ*_1_ = μ _2_)*.

**Figure 4:**
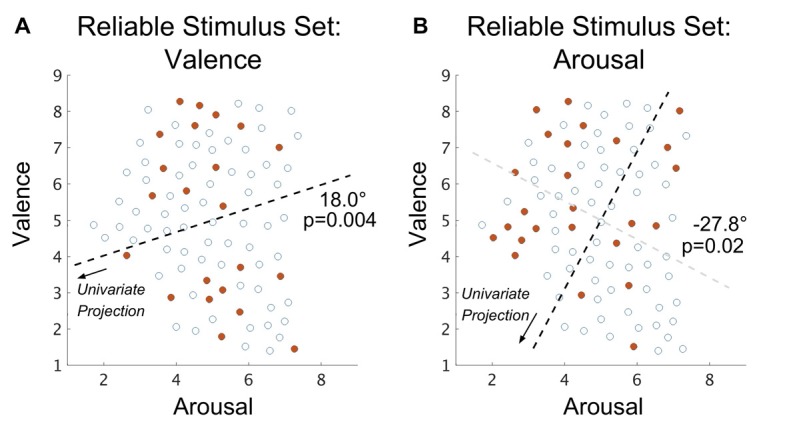
Spatial distribution (within the arousal-valence plane of normative affective scores) of those IAPS stimuli that exhibit group-level classification accuracy that is significantly better than chance in the case of **(A)** valence property classification and **(B)** arousal property classification. Open circles represent the normed perceived affect coordinates of the full stimulus set (FSS) stimuli that are not part of the reliable stimulus set (RSS). Closed red circles represent the normed affect coordinates of the joint RSS evaluated over all subjects. Dark dashed lines depict the axes of significant multimodality (bootstrap method, *α* = 0.05); arrows indicate the directions of projection of the affect scores forming the univariate distributions on which tests of multimodality were conducted. The angle reported is the rotation (referenced from clockwise) of the base axis necessary to achieve the multimodal axis. The light gray dashed line depicted in **(B)** denotes the rotation; however, as arousal coordinates are orthogonal to valence coordinates the axis of univariate projection is orthogonal to this line.

According to the methodology outlined in Figure 1E (see “Materials and Methods: Intra-subject Stimulus Subset Selection” section for details), for each intra-subject classification analysis we selected only those IAPS stimuli that exhibited group-level accuracy (over the *n* = 18 subjects not part of the intra-subject classification) that was significantly greater than chance (null hypothesis is the binomial distribution, *n* = 18, *p* = 0.5, *α* = 0.05), which we term the RSS. Specific stimuli of the RSS are plotted in relation to the FSS in Figure 4. We then conducted intra-subject classification of these stimuli (relying on the SVM hyperplane learned from the FSS). The results of this classification experiment are summarized in Table 1 (RSS cells). Classification accuracy in this case significantly exceeds that of the FSS for the properties of valence, arousal and overall AS. Moreover, subject-wise, rather than group, classification analysis found 19 of 19 (100%) individuals’ brain states classified valence significantly greater than chance and 19 of 19 (100%) individuals’ brain states classified arousal significantly greater than chance (null hypothesis for both tests is the binomial distribution, *n* = |RSS_i_|, *p* = 0.5 and *α* = 0.05, where RSS_i_ denotes the RSS constructed for subject (i). Overall, these group-level and subject-wise findings were comparable to those reported by Baucom et al. (2012).

### Affective Properties of the Stimulus Set Characterize the Reliability of Classifications

We explored the stimuli selected for inclusion the RSS. Using simulations based upon group mean classification accuracies to construct null distributions of the RSS set sizes, respectively for valence and arousal (see “Materials and Methods: Simulation of Chance Subset Proportion” section), we found that the size of the observed RSS is significantly larger than what would be expected by chance for both valence (*p* < 0.001; bootstrap method) and arousal (*p* < 0.001; bootstrap method). This suggests that passive viewing of the RSS induces brain states (across the entire group of subjects) that are biased toward classifying an affective property. To control for variation in the IAPS normative scores, we compared the standard deviations of the IAPS normative scores of the RSS with those stimuli within the FSS that did not exhibit group-level accuracies different from chance. This comparison found no significant differences in stimulus variance for valence (*p* = 0.62; 2-sample *t*-test, *α* = 0.05; null: *μ*_1_ = *μ*_2_) nor arousal (*p* = 0.49; 2-sample *t*-test, *α* = 0.05; null: *μ*_1_ = *μ*_2_).

We also explored whether the arousal-valence properties of RSS stimuli were related to their inclusion in the RSS. Based on the classification performance reported by Baucom et al. (2012), we hypothesized that membership in the RSS would be characterized, respectively for valence and arousal, by bimodal distributions of extreme values. Past work involving the IAPS (Bradley et al., 2001), however, suggests collinearity between the valence and arousal properties of affect that cannot be easily separated. To accommodate prior work we rotated the arousal-valence plane (independently for valence- and arousal-derived RSS imagesets), identified the Hartigan dip test statistic (Hartigan and Hartigan, 1985) maximizing angle, and tested the rotated imagesets’ properties for multimodality via the bootstrap method (see “Materials and Methods: Test of Spatial Multimodality” section). We found that, indeed, both the valence-derived RSS (max angle = 18.0°; *p* = 0.004; bootstrap method); and the arousal-derived RSS (max angle = −27.8°; *p* = 0.02; bootstrap method) exhibited significant bimodality.

### Brain State Encoding of Autonomic Arousal Is Affect Stimulus Set-Dependent

**Figure 5:**
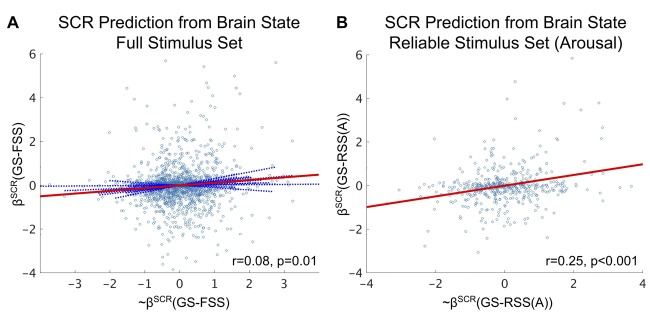
Brain state predictions of affective arousal as measured by *z*-scored SCR. **(A)** SVM-predicted SCR state significantly predicts the true SCR state over the FSS (fixed effect: *r* = 0.08, *p* = 0.01, *F-test*). **(B)** SVM-predicted SCR state significantly predicts the true SCR state over the arousal-derived RSS (fixed effect: *r* = 0.25, *p* < 0.001, *F-test*). Circle markers indicate individual stimuli of the study. Red lines depict fixed-effects. Solid blue lines indicate significant random effects. Dashed blue lines indicated insignificant random effects.

As depicted in our conceptual model, we hypothesize that there exists a one-to-one mapping between the affective content of each image stimulus and its fMRI-derived brain state. For validity, we concurrently measured autonomic arousal responses via SCR during fMRI identification of the affective brain states. As part of our analysis, we fit intra-subject SVM regression models of SCR states (beta-values) from our fMRI-derived brain states and compared (via GLMM) the agreement between our model predictions and ground truth when the model incorporated either the FSS or the arousal-derived RSS. Similar to the vastly improved accuracy obtained by limiting classification to the RSS vs. the FSS, model-based SCR prediction effect size (Pearson’s *r*) is improved more than threefold when restricted to the RSS, depicted in Figure 5.

While significant, our fMRI-derived brain state predictions of SCR states based on the FSS (see Figure 5A) exhibit only small effects (Cohen, 1992). It is possible that the effectiveness of these predictions is limited by the failure of some stimuli to elicit a significant change in the SCR. We explored this possibility by conducting a median split of the SCR states (keeping only SCR states greater than the median to simulate the elicitation of strong SCR). We then recomputed the effect size of the fMRI-derived brain state predictions on this subset and found that prediction effect size doubled (fixed effect: *r* = 0.16, *p* = 0.007, *F*-test).

### Affective Brain States Significantly Disagree With Normative, Self-Reported Experiential Affect

Similar to the methodology employed to select the RSS, we also formed an image subset based on only those stimuli that exhibited group-level accuracy (over the *n* = 18 subjects not part of the intra-subject classification) significantly worse than chance (null hypothesis is the binomial distribution, *n* = 18 (subjects), *p* = 0.5, *α* = 0.05), which we term the USS. We then conducted intra-subject classification of these stimuli (relying on the SVM hyperplane learned from the FSS). The results of this classification experiment are summarized in Table 2; classification accuracy in this case significantly subceeds that of the FSS for both the valence and arousal properties of dimensional affect. Comparisons of USS to FSS for overall AS were not possible due to an empty set of surviving stimuli.

**Table 2:** Intra-subject classification performance of the Incorrect Stimulus Set, analyzed for group-level significance.

	Valence (V)	Arousal (A)	Affective state (AS)
	Grp. Avg. Acc. (95% CI)	Grp. Avg. Acc. (95% CI)	Grp. Avg. Acc. (95% CI)
Unreliable stimulus set (USS)	0.21^†‡^ (0.16, 0.26)	0.33^†‡^ (0.23, 0.43)	—*^♠^*

*^†^Finding is significantly worse than chance accuracy (2-sided 1-sample t-test, *α* = 0.05; null_V,A_: μ = 0.50). ^‡^Accuracy is significantly worse than FSS accuracy (2-sided 2-sample t-test, *α* = 0.05; null: *μ*_1_ = *μ*_2_). ^♠^No stimuli were captured by the group-level selection criteria*.

**Figure 6:**
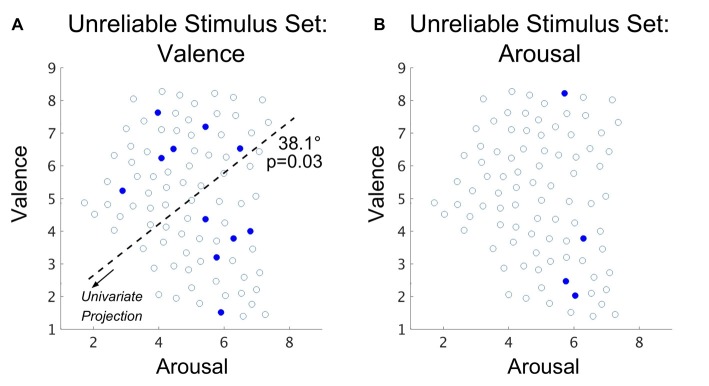
Spatial distribution (with the arousal-valence plane of normative experienced affective scores) of IAPS visual stimuli that exhibit group-level classification accuracy that is significantly worse than chance in the case of **(A)** valence property classification and **(B)** arousal property classification. Open circles represent the normed perceived affect coordinates of the FSS stimuli that are not part of the USS. Closed red circles represent the normed affect coordinates of the joint USS evaluated over all subjects. The dark dashed line depicts the axis of significant multimodality (bootstrap method, *α* = 0.05); the arrow indicates the direction of projection of the affect scores forming the univariate distribution on which a test of multimodality was conducted. The angle reported is the rotation (referenced from clockwise) of the base axis necessary to achieve the multimodal axis.

Specific stimuli of the USS are plotted in relation to the FSS in Figure 6 for both valence and arousal classifications. In line with our analysis of the RSS, we explored the distribution of stimuli selected for the USS. Using simulations based upon group mean classification accuracies to construct null distributions of the USS set sizes, respectively for valence and arousal (see “Materials and Methods: Simulation of Chance Subset Proportion” section), we found the size of the USS is significantly larger than what would be expected by chance for the valence property (*p* < 0.001; bootstrap method) but not for the arousal property (*p* = 0.40; bootstrap method); therefore, distribution analysis of arousal-derived USS was not conducted. This finding suggests that brain states that incorrectly encode (over the entire group) the normative valence property of affect comprise 12.2% of the total stimulus set.

As a check, we compared the standard deviations of the normative valence scores between the USS and the subset of FSS stimuli that did not exhibit group-level accuracies different from chance. We found no significant group differences (*p* = 0.94; 2-sample *t*-test, *α* = 0.05; null: *μ*_1_ = *μ*_2_). Similar to our analysis of the RSS, we also tested the valence-derived USS for multimodality to determine whether these classification errors may be driven by a specific region of the arousal-valence plane. The Hartigan dip test statistic was maximized by a 38.1° of the arousal-valence plane rotation (i.e., approaching equiproportional blend of valence and arousal properties), producing significant multimodality (*p* = 0.03; bootstrap method).

## Discussion

### The Role of Stimulus Selection in Multivariate Affect Prediction Performance

The central aim of this research was to understand cross-study differences in the multivariate analysis of affect processing literature. We did so through the lens of the brain state hypothesis of affect processing, which posits a one-to-one mapping between each affective stimulus and its fMRI-derived, temporally succinct pattern of neural activation. As the third exploration of MVPA-based classification of dimensional affect using comparable methodology (previously Baucom et al., 2012; Bush et al., 2017), the novel approach put forth in this study was a data-driven evaluation of the limits of affect property classification performance that can be achieved via IAPS image-induced affect perception.

The intra-subject classification accuracies achieved by our modeling approach within the RSS are comparable to the previously best reported findings for the classification of perceived valence, arousal, and combined AS (Baucom et al., 2012). Moreover, analysis of the spatial distribution of RSS images within the arousal-valence plane identified significant image clusters (relative to the FSS) at the extremes of low-arousal-positive-valence and high-arousal-negative-valence, a novel finding. Combined, this evidence supports the hypothesis that the clustered image stimuli selected by Baucom et al. (2012) significantly contributed to their reported classification performance. Based on accuracies achieved via our data-driven stimulus selection method, we quantify the impact of this stimulus selection bias to be on the range of 23%–35% in reported accuracy.

The intra-subject affect classification accuracies that we report for the FSS (for both valence and arousal classification) fall significantly between previously reported classification accuracies: significantly worse than Baucom et al. (2012) and significantly better than the intra-subject classification accuracies reported by Bush et al. (2017). Given the methodological similarity of these three studies, this performance gap suggests the existence of an unaccounted variable beyond stimulus selection—specifically, the differences between the Bush et al. (2017) study and the findings reported here. A methodological review of these studies found that the critical difference not explained by the spatial distribution of stimuli within the arousal-valence plane is the total number of stimuli presented to each subject: Bush et al. (2017) presented *n* = 45 stimuli per subject; the FSS portion of this study presented *n* = 90 stimuli per subject; and, Baucom et al. (2012) presented *n* = 80 stimuli per subject.

To assess the role of the quantity of affective stimuli as a confound to classification performance, we conducted *post hoc* experimental analysis in which we created stimulus sets of varying sizes (25%–100% of total stimuli, increasing at increments of 5%, sampled uniformly randomly from the FSS) and conducted intra-subject LOOCV classification. Then, separately for valence and arousal, we fit GLMMs to the results, using classification accuracy as the measure of interest and stimulus set size as the fixed effect. Random effects were modeled subject-wise. The result of these tests (see Supplementary Figure S2) show stimulus set size to have a significant moderate effect (*r* = 0.20 and 0.31, respectively for valence and arousal) on classification accuracy. This finding supports the methodological convention of using a large image stimulus set to characterize affect processing. However, methodology must also be mindful of competing constraints: the participant’s comfort, ability to maintain attention, as well as potential stimulus degradation due to habituation.

### Validating the Brain State Hypothesis

By testing cross-study differences in the classification performance of neural encodings, we developed strong evidence in support of the brain state hypothesis of affect processing. For the first time in the literature of MVPA-based affect classification, we simultaneously demonstrated inter-study reliability of our models as well as convergent validity of our model parameters and predictions with prior prediction outcomes reported in the multivariate affect prediction literature, neuroanatomical findings reported in the univariate affect encoding literature, as well as established relationships between autonomic arousal and the SCR reported in the psychophysiology literature.

We also identified encodings of affect processing that exhibit non-canonical involvement of the executive network in affect processing (see Figure 2, insets). Specifically, right ventral lateral prefrontal cortex, right dorsal lateral prefrontal cortex, and right posterior middle temporal gyrus were activated across the affective encoding of both valence and arousal properties in a manner that is consistent with a preparatory/modulatory response to evaluated threat (negative valence and high arousal), but encoded by regions associated with emotion regulation (Etkin et al., 2015), rather than perception. This finding may suggest that the brain state hypothesis extends to additional aspects of emotion processing.

Finally, an important component of this study was the validation of brain states as central affect encoding units evidenced by their significant prediction of an independent measurement, autonomic arousal, captured via SCR. The threefold increase in this prediction’s effect size accompanying the restriction of prediction to the arousal-derived RSS suggests an atypically high degree of coupling between brain state and autonomic response in the RSS. While much of this coupling may be explained by controlling for strong SCRs, approximately 56% of this increased effect size remains unexplained.

### Challenging the Brain State Hypothesis

Our study also identified the valence-derived USS, a subset of the FSS containing stimuli that induced group-wide brain states that were misclassified significantly more often than chance. The presence of the USS would seem to invalidate the brain state hypothesis; however, we propose two possible explanations for the existence of the USS that potentially preserves its validity. Each brain state in our experiment is classified with respect to other brain states of a specific subject. During model fitting, it is possible for voxels to encode stimulus properties that are correlated with valence (imagine, e.g., the visual consistency of wounded humans in negatively valent stimuli or baby faces in positively valent stimuli). These properties may be used to inform the classification label. If the training set is sufficiently biased to a correlated property, then stimuli of similar affective character that do not exhibit the property could be consistently mislabeled. If our stimulus set contains this bias, then our experiment could produce the USS while remaining consistent with the brain state hypothesis. An alternative explanation of the USS is that its normative valence scores incorrectly reflect the perceived affective content of the stimulus. Because the scores are normed over many subjects, this explanation would be possible only if the scores were biased either by the population or the environment from which they were sampled.

We have found evidence to support both of these possible explanations. Due to user agreement, we cannot publish the IAPS images comprising the FSS, RSSs and USSs; however, we have published the full set of IAPS image IDs used to construct the FSS (see Supplementary Table S1) as well as the short text descriptions of the images comprising the reliable and unreliable image subsets separately for both valence and arousal classifications (see Supplementary Table S2). The value of these text descriptions is that they suggest biases in the RSS (toward infants/children, wounds, and erotica) that may reflect biases in the portions of the brain states selected by the classifier during training, which are not reflected in stimuli of the USS. We also reviewed the original IAPS development methodology and found that subjects rated the images in large groups, viewed each stimulus for 6 s, and rated the image for 15 s along the three primary dimensions of affect (Lang et al., 2008), the third being dominance. This acquisition methodology, which differs significantly from the environment in which we apply the normative scores, may have biased (e.g., through social processing) the normative scores.

### Limitations and Future Directions

This work translates a series of important affect processing findings into methodological recommendations for future studies of fMRI-derived MVPA-driven neural pattern classification of dimensional affect, specifically, over-sampling stimuli from the affective extreme, group-level validation of the induced brain states with respect to normative scores of perceived affect, as well as verification of the fMRI-derived brain states with respect to independent measures of affects. However, the precise functional neuroanatomical structure of affect processing brain states remains unclear.

A limitation of our approach is evident in our attempt to reproduce the brain state encoding of valence. While highly significant and exhibiting moderately large effect size, inter-study prediction explains only approximately 15% of the valence encoding’s variance. Clearly some, but not all, of this variance is attributable to individual encoding differences between the two sample populations. Future work, incorporating hyperalignment methodology (Haxby et al., 2011), could be used to control for those differences.

It is also worth highlighting that the EAP encoding parameters were derived from inter-subject LOOCV predictions whereas the INCA encoding parameters were derived from intra-subject LOOCV predictions, a potential source of variance (intra-subject predictions were not found to be significant in the EAP study due to the small number of stimuli per subject; therefore, the direct comparison was not made). We also note that fMRI data for some subjects (*n* = 18) within the EAP study were acquired using an 8-channel headcoil, whereas the remaining subjects’ data were acquired using the same 32-channel headcoil used for all INCA subjects, another potential source of variance.

We also attribute unexplained variance in the valence encoding to the manner in which stimuli were sampled between the EAP and INCA studies. EAP stimuli were sampled uniformly randomly from IAPS (see Bush et al., 2017), which artificially correlates high valence (both positive and negative) stimuli with high arousal, likely biasing the EAP’s valence encodings to include regions simultaneously encoding arousal. Evidence for this comes from the fact that inter-study prediction explains 55% of arousal encoding variance, which is not subject to this correlation (e.g., a given valence-encoding voxel would only correlate with arousal for 50% of stimuli). A future replication study, employing a maximum separation-based sampling of IAPS stimuli but conducted at an independent site (i.e., different scanner, personnel and analysis source code) would serve as a true inter-study reliability test of affective encodings.

We also validate our models against only one independent representation of affect, autonomous arousal, measured via SCR. The authors are currently engaged in large-scale studies of affect processing and regulation in which complementary psychophysiological and behavioral measures of both the valence and arousal properties of affect (Bradley et al., 2001) are acquired in conjunction with fMRI.

Finally, future studies should incorporate design elements that test (or control for) our hypothesized explanations of the existence of a significant USS, the presence of which suggests limitations in our stimulus set either through confounded properties or confounded labels. Controlling for confounding properties requires meticulous diversification of stimuli in the arousal-valence plane to minimize the likelihood of one or more extraneous stimulus properties correlating with valence or arousal. To control for possible confounds in the normative scores, we suggest re-scoring the IAPS imageset under conditions analogous to how the images are applied in this and other fMRI-based studies of affect processing (the subject should be isolated and tasked with scoring following very brief image presentation duration (≤2000 ms)).

## Author Contributions

KB designed the INCA study and implemented the analytical experiments. JG designed and implemented the dimensionality reduction processing pipeline and made critical contributions toward constructing a null hypothesis of the reliable and unreliable stimulus sets. AP designed and validated the psychophysiological processing pipeline. M-HC enforced the quality of the statistical analysis of the manuscript. GJ designed, implemented and collected the affective data of the EAP study. GJ and CK enforced the quality and relevance of the manuscript. KB drafted the manuscript with critical revisions by AP, M-HC, GJ and CK.

## Conflict of Interest Statement

The authors declare that the research was conducted in the absence of any commercial or financial relationships that could be construed as a potential conflict of interest.

## Supplementary Material

The Supplementary Material for this article can be found online at: https://www.frontiersin.org/articles/10.3389/fnhum. 2018.00262/full#supplementary-material
