# Supplementary material for: Brain States That Encode Perceived Emotion Are Reproducible but Their Classification Accuracy Is Stimulus-Dependent
Source: Front Hum Neurosci. 2018 Jul 2;12:262. doi: 10.3389/fnhum.2018.00262 (PMC6036171; doi:10.3389/fnhum.2018.00262)
Supplement: Supplementary file 1 [file Data_Sheet_1.DOCX]

Supplementary Material

**Brain States that Encode Perceived Emotion are Reproducible but their Classification Accuracy is Stimulus-Dependent.**

**Keith A. Bush*, Jonathan Gardner, Anthony Privratsky, Ming-Hua Chung, G. Andrew James, Clinton D. Kilts**

***Correspondence:** Keith A. Bush, kabush@uams.edu

# Supplementary Figures and Tables

**
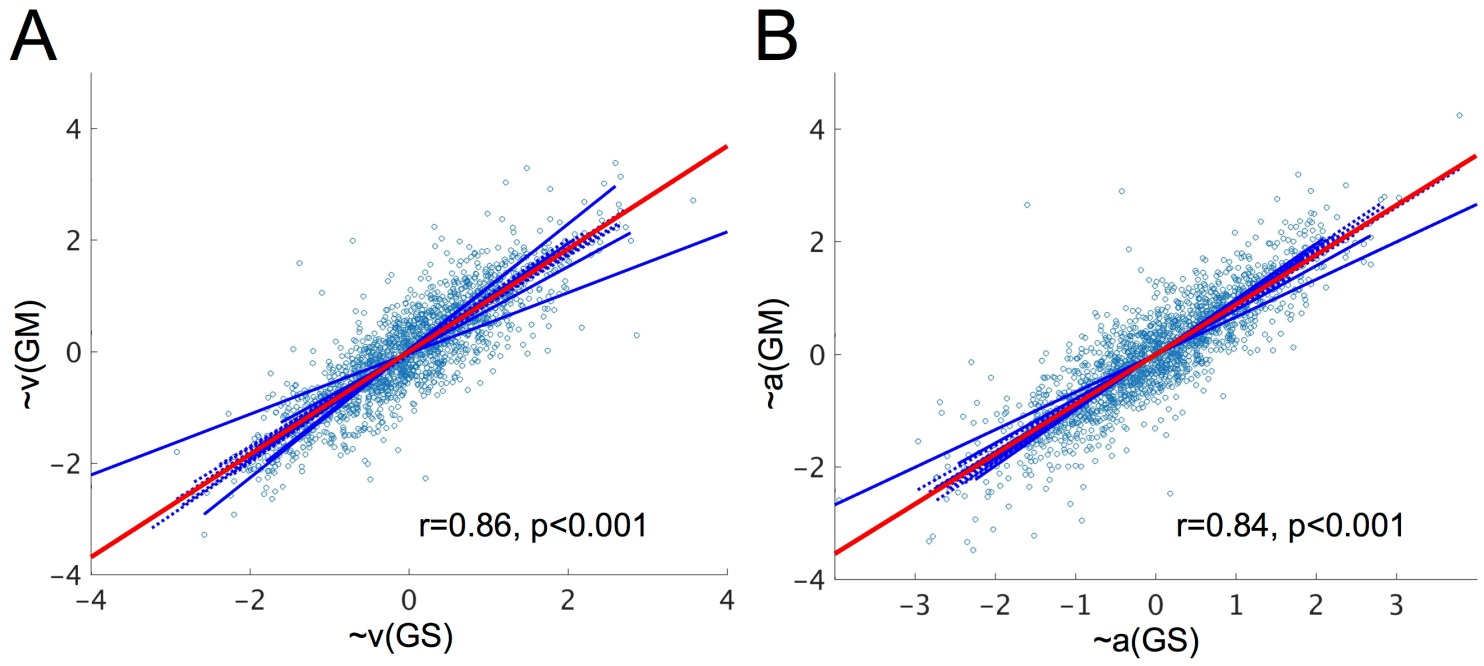
**

**Supplemental Figure 1**: SVM estimated hyperplane distances based on a Gram-Schmidt reduced dimensional features strongly predict SVM estimated hyperplane distances based on whole-brain gray-matter features according to a general linear mixed-effect model (GLMM). Random effects were modeled subject-wise. (A) Valence predictions (fixed effect: r=0.86, p<0.001, F-test). (B) Arousal predictions (fixed effect: r=0.84, p<0.001, F-test).


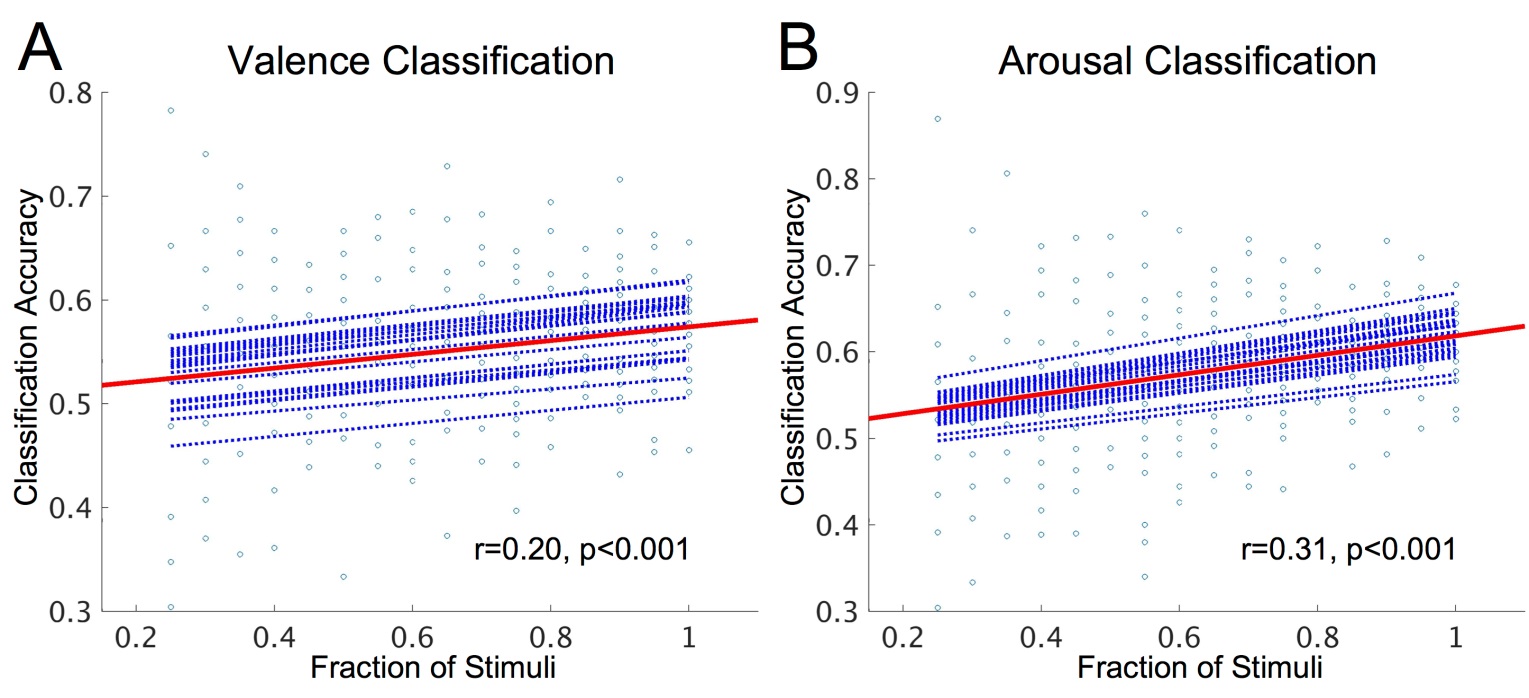


**Supplemental Figure 2**: GLMM model of classification accuracy as a function of stimulus subset size on which the classifier was trained. Measure of interest was intra-subject classification accuracy. Predictor was the size of the stimulus subset as a fraction of the size of the FSS. Random effects modeled subject-wise. **(A)** Affective valence classification accuracy as a function of stimulus subset size (fixed effect: r=0.20; p<0.001, F-test). **(B)** Affective arousal classification accuracy as a function of stimulus subset size (fixed effect: r=0.31; p<0.001, F-test).

| **Extrinsic Format IAPS Image IDs** |
| --- |
| 1750, 5982, 2540, 9120, 4641, 7100, 2520, 1050, 4503, 9832, 9360, 7175, 4619, 3310, 1022, 2020, 6300, 7211, 9331, 9622, 4531, 3250, 9254, 8475, 4626, 9435, 1931, 4800, 7043, 7285, 9163, 2279, 5750, 2058, 4770, 8160, 2722, 3500, 4550, 7490, 3015, 2205, 7031, 2351, 6930, 4598, 2040, 7492, 5950, 4597, 5010, 2095, 8158, 2217, 2352, 8190, 1301, 9415, 9184, 5760, 1620, 5833, 6550, 5395, 8200, 3102, 6563, 2222, 9220, 4490, 5020, 4235, 8186, 7217, 9102, 3000, 2271, 1333, 9426, 9700, 4649, 2302, 8231, 4220, 2795, 7480, 7224, 1810, 9390, 8030 |

**Supplemental Table 1:**  IAPS image IDs for the full stimulus set (FSS).

| **Reliable Stimulus Set (RSS)** | | **Unreliable Stimulus Set (USS)** | |
| --- | --- | --- | --- |
| **Valence Derived** | **Arousal Derived** | **Valence Derived** | **Arousal Derived** |
| Bunnies  Sky  Snake  EmptyPool  Romance  Adult  HomelessMan  EroticMale  Wedding  Baby  Baby  Toddler  Dog  Handicapped  InjuredDog  Antelope  EroticFemale  Skysurfer  Heroin  Mutilation  Assault  ChildCamera | Bunnies  Sky  OilFires  Romance  FireHydrant  Cigarettes  EmptyPool  Incubator  Clock  Biking/train  EroticCouple  FemaleKiss  Accident  Shoes  Class  Nature  Antelope  Boat  BoysReading  Flower  Skysurfer  ClothesRack  Trash  EroticFemale  FileCabinets | Mother  OilFires  Romance  FireHydrant  Incubator  OpenChest  Shark  Accident  Hiker  Class  Hippo | OpenChest  Assault  InjuredDog  Beach |

**Supplemental Table 2:** IAPS short text descriptions of reliable stimulus subsets (RSS) and unreliable stimulus subsets (USS), separated, respectively, by affective property (valence and arousal).
